# Issues Related to Sentinel Lymph Node Assessment in the Management of Breast Cancer—What Are Relevant in Pathology Reports?

**DOI:** 10.4061/2011/504940

**Published:** 2011-03-15

**Authors:** Patricia Tai, Kurian J. Joseph, Edward Yu

**Affiliations:** ^1^Department of Radiation Oncology, Allan Blair Cancer Center, SK, Canada S4T 7T1; ^2^Department of Oncology, Cross Cancer Center, AB, Canada T6G 1Z2; ^3^Radiation Oncology Division, London Regional Cancer Program, ON, Canada N5A 4L6

## Abstract

Most cancer centers now perform sentinel node (SN) biopsies. The limited number of SNs sampled compared with an axillary dissection has allowed more comprehensive lymph node analysis resulting in increased detection of micrometastases. Many node-negative cases are now reclassified as micrometastatic. Recent research on SN biopsy focuses on whether axillary dissection is always necessary when the SN is positive. Some subgroups of patients have a higher risk of more nodal metastases when completion axillary dissections were performed. This paper summarizes the different studies and examines what are the clinically relevant items to report on SN node pathology: volume or size of nodal metastasis, location within the node, extranodal extension, number of involved SN(s) and non-SN(s), total number of SN, and total number of nodes on axillary dissection, if performed.

## 1. Introduction

Breast cancer is estimated to have an incidence of 22,700 and will cause 5400 cancer deaths among females in Canada in 2009 as per Canadian Cancer Society Statistics [1]. Better prognostic factors to aid oncologists in making treatment decision will benefit a significant number of patients. The nodal staging of breast cancer generally involved a level I and II axillary dissection. To reduce the risk of surgical complications, sentinel node (SN) biopsy has been widely used in the last decade. 

*Nodal ratio* (absolute number of involved nodes/number of nodes resected) was recently proposed to have a greater prognostic value than absolute number of involved nodes [2–7]. Since the paper by Woodward et al. [8] from the International Nodal Ratio (NR) Working Group, there are a few more studies confirming this [9]. In a recent study, relapse free and overall survival rates were not different according to the absolute number of involved nodes (*P* = .166, *P* = .248, resp.) [10], but on multivariate analysis, the NR was an independent prognostic factor for relapse free and overall survival (Hazard ratio, HR = 4.246, *P* < .001; HR = 7.764, *P* < .001), respectively.

*Different dividing lines for NR* have been used in the literature. Our previous work showed a survival benefit for regional nodal radiotherapy (RT) when the NR of axillary nodes is 0.25 or more [11]. In this study, patients were categorized into three NR groups; low (LNR, ≤25%), medium (MNR, >25% to ≤75%) and high (HNR, >75%) nodal involvement. This categorization follows previous literature using British Columbia data [2] and American data [8, 12]. Truong et al. found that 25% is a good dividing line for grouping [2]. 

With *sentinel node (SN) mapping* technique, the minimum number of nodes required for accurate staging becomes less. This is because SN biopsy technique uses radio-isotope and dye to guide the search for first drainage node(s) accurately. SN biopsy correctly identifies the involved node which could be missed by axillary node dissection without any guidance [13]. Analysis of frozen section of SNs is an accurate method for metastasis detection, allowing axillary dissection when positive at the same operative setting [14]. 

 Controversies of sentinel node assessment abound. This paper aims to *summarize and analyze* the current management of breast cancer following SN biopsy. Recommendations to target readers (clinical oncologists and pathologists) are suggested.

## 2. Material and Methods

A search of PubMed and the proceedings of the American Society for Therapeutic Radiology and Oncology (ASTRO) and American Society of Clinical Oncology (ASCO) annual meeting books was performed and selected relevant articles and abstracts pertinent to SN assessment and prognostic relevance.

## 3. Results and Discussion

The limited number of SNs compared with an axillary dissection has allowed more comprehensive lymph node analysis resulting in increased detection of micrometastases. Many women previously classified as node-negative are now reclassified as having micrometastatic nodal involvement. As a result, our nodal classification and cancer staging have evolved to recognize the continuum of nodal tumor burden rather than a simplistic dichotomous stratification [15]. The pathologist is expected to mount, stain, and microscopically examine serial sections of the SN using hematoxylin and eosin (H&E) staining. Despite recommendations from the College of American Pathologists and the American Society of Clinical Oncology, heterogeneity in the approach to SN evaluation exists. What is needed is adherence to a standardized evaluation protocol. The most important aspect of the SN examination is careful attention to slicing the SN with thickness no more than 2.0 mm and correct embedding of the slices to assure all macrometastases larger than 2.0 mm are identified.

### 3.1. Is Minimal Lymph Node Involvement Clinically Relevant? 

There is an ongoing debate concerning the clinical implications of micrometastases in the SN. Many observational studies have been published but results do not justify conclusions. Bulte et al. [18] of Netherlands looked at the subgroup of patients with micrometastases (*n* = 38) :  3 (7.9%) patients developed distant recurrence. In the group with a tumour-free sentinel node (*n* = 503), 17 (3.4%) distant recurrence and 3 (0.6%) combined regional and distant recurrence were observed. The rates of distant recurrence between the node-negative and micrometastatic cases are not significantly different (Chi-square test, *P* = .128). However, the authors reported that the result may be limited by small sample size. Despite the lack of statistical significance of outcome of pN1mi in reference 18, to an individual patient the worse outcome is still *clinically important*.

Indeed other studies show that the prognosis of patients with pN1mi is significantly worse compared to node-negative patients, in terms of regional and distant recurrence rates [19]. The worse prognosis was further confirmed by a large SEER database study [20]: breast cancer specific survival (BCSS) and overall survival with pNmi disease progressively declined with increasing number of positive nodes and increasing NR.

In the MIRROR (Micrometastases and ITCs: Relevant and Robust or Rubbish?) study, almost all participating pathology laboratories used a protocol in which the SN was serially sectioned at least every 150 *μ*m and at a minimum of three levels, with the use of keratin immunohistochemical (IHC) staining if the H&E staining was negative. In contrast, the nonSNs were macroscopically sectioned every 2 to 5 mm, and one section per slice was stained with H&E. The aim was to evaluate the relationship, if any, between ITCs or micrometastases in the regional lymph nodes and clinical outcome in patients who had undergone an SN procedure and who did or did not receive systemic adjuvant therapy. *They found that adjuvant treatment helped to lower the risk of disease events* [21]. The median followup was 5.1 years. This agrees with large studies that included women who received a diagnosis before the SN era; micrometastases, defined as 2 mm or smaller in diameter and including ITCs, were associated with reduced overall survival [22–25]. In these studies, however, the axillary nodes were examined by means of H&E staining at just one level. Thus, we cannot compare these studies with the MIRROR study, which involved a detailed examination of the SN. The few previous studies of SNs were limited by small samples, lack of multivariate analyses, or short followup [26–28]. 

It is noteworthy that for patients with minimal nodal involvement, the disease-free survival (DFS) was *initially similar but started to fall after the third year compared to node-negative results* [29]. Patients with pN1a and pN1mi/pN0i+, when compared with patients with pN0 disease, were more often prescribed anthracycline-containing chemotherapy (39.1% versus 33.2% versus 6.1%, resp., *P* < .0001) and were less likely to receive endocrine therapy alone (9.8% versus 19.4% versus 41.9%, resp., *P* < .0001). On multivariate analysis, a statistically significant difference in DFS and in the risk of distant metastases was observed for patients with pN1a versus pN0 disease (HR = 2.04; 95% CI, 1.46 to 2.86; *P* < .0001 for DFS; HR = 2.32; 95% CI, 1.42 to 3.80; *P* = .0007 for distant metastases) and for *patients with pN1mi/pN0i+ versus pN0 disease *(HR = 1.58; 95% CI, 1.01 to 2.47; *P* = .047 for DFS; HR = 1.94; 95% CI, 1.04 to 3.64; *P* = .037 for distant metastases). 

In summary, Table 2 shows that pN1mi patients consistently have an HR for events of 1.5 versus node-negative patients. Hence despite smaller studies with shorter followup showing no significant difference in outcome [18, 30], with the best available evidence at the present time, the authors of this paper felt that pN1mi patients tend to have worse outcome than node-negative patients. 

In our institute, medical oncologists tend to treat patients with micrometastases (0.2–2 mm node) with adjuvant systemic treatment, while the treatment for nodal metastases <0.2 mm is still debatable. For patients with nodal metastasis ≤2 mm including ITC, the use of nodal radiotherapy is controversial. A muticenters trial for these patients with enough followup duration, and to stratify tumor size, grade and nodal ratio may provide further insight to the role of nodal radiotherapy. 

### 3.2. Completion Axillary Dissection after a Positive SN Biopsy

Another area of recent research on SN biopsy focuses on whether axillary lymph node dissection (ALND) is always necessary when the SN is positive [31]—what is the probability of further nodal metastases in the axilla? Here we examine the available research studies on this issue. 

A study of 159 stage T1 or T2 breast cancer patients speculated that axillary dissection *can be avoided in those patients diagnosed of micrometastasis* in the SN [32]. Completion ALND was performed when micro or macrometastases were found in the SN. A total of 40 patients (25%) showed infiltration of the SN. This infiltration was only by micrometastasis in 17 of them (10.7%). Of these 17 patients, only 2 *(11.8%)* showed macrometastasis in the lymphadenectomy specimens. 

#### 3.2.1. Which Patients Can Be Safely Selected to Forgo Completion ALND? 

Table 2 shows that if micrometastasis is found in a SN, omission of additional ALND may be envisaged by Houvenaeghel et al. with minimal risk for pT1a and pT1b tumors, and pT1a-b-c tumors corresponding to *tubular, colloidal, or medullary* cancers [33]. 

A study from São Paulo of 1,000 successive patients with SN biopsy from 1998 to 2008 put this issue into context [34]. The mean age was 57.6 years and mean tumor size was 1.85 cm. A total of 72.2% SN were negative and 27.8% were positive, but in 61.9% of the cases, the SN was the only positive one, with 78.4% having macrometastases, 17.3% micrometastases and 4.3% ITCs. After 54 months of followup, there were no recurrences in patients with ITCs, but one local recurrence and two systemic recurrences were observed in the micrometastases group, as well as four local and 30 distant metastases in the macrometastases group. *Among the clinical parameters studied, only tumor size was correlated with metastatic involvement in axillary lymph nodes.* The size of the metastases and the number of positive SN also directly increased the possibility of systemic recurrence. 

*Volume of disease* in the SN is a significant predictor of additional nodal metastasis. In a Memorial Sloan-Kettering Cancer Center study of 505 patients, 251 pN0(i+) and 254 pN1mi: 12% of pN0(i+) and *20%* pN1mi had additional nonSN disease [35]. On multivariate analyses including eight variables, only *lymphovascular invasion* (odds ratio > 2.2, *P* < .01) and *volume of nodal metastasis* as assessed by any method of measurement (method of detection, AJCC, and cell count) were significantly correlated with additional nonSN disease (*P* = .04,   .03,  and .02, resp.). 

More pathologic risk factors were investigated in another study of 128 patients who had a positive SN biopsy in 2005–2007 [36]. The metastases in each SN were assessed according to their location within the node (subcapsular, mixed subcapsular and parenchymal, parenchymal, multifocal or extensive) and metastatic infiltration of perinodal tissue was also reported. The strong predictors of the axillary lymph node metastasis included the SN metastasis diameter (7.6 versus 4.4 mm) and size classified according to WHO classification (ITC 0 versus 100%, micrometastasis 23.5 versus 76.5%, macrometastasis 51.9 versus 48.1%). The *SN metastases with a diameter of above 3* *mm* were associated with approximately twice more frequent ALN metastases. If there is extensive SN metastasis, the highest percentage of ALN metastases was found (65 versus 35%). The weak predictors of ALN metastases were: primary tumor diameter (>2 cm), immunohistochemical HER2 positive status, infiltration of sentinel perinodal tissue by metastasis, histological primary tumour grade. 

Two other important concepts to select patients for completion ALND to mention are nomograms and nodal ratio. *Nomograms* or other scoring systems have been used to predict the chance of involvement of nonSNs after a single involved SN is found [37, 38]. The *nodal ratio* concept has been extended to SN biopsy. More than one positive SN and a ratio of positive SN(s) to total SN(s) of greater than 0.5 were found to be predictors for additional axillary nodal involvement in both univariate and multivariate analyses [39]. The number of positive SNs and the SN nodal ratio is an indication of total tumor burden in the SNs and may be a reflection of the propensity of the tumor for further lymphatic invasion in the axillary basin.

#### 3.2.2. What Is the Significance of IHC Positivity in SN Which Is H&E Negative? 

The surgeons at the St Vincent's University Hospital in Dublin, Ireland performed SN mapping for breast cancer from January 1st, 2000 to December 31st, 2006 [40]. All SNs were assessed by serial H&E and IHC sections. Patients with micrometastases (0.2–2 mm) underwent completion ALND. Patients with ITC (<0.2 mm) were individually discussed and an ALND was performed selectively based on additional clinicopathological criteria and patient preference. Patients were followed for a median of 27 months (range 12–72 months). 1076 patients who underwent SN were included for analysis. The experience is unique as it demonstrates the breakdown of cases into each category: 211 (20%) had a positive SN biopsy using H&E. *Forty-nine patients (5%) had a negative SN on H&E which was positive on IHC.* Of these, *15 had micrometastases and underwent an ALND. Two had further axillary nodal disease.* ITC were found in the remaining 34 patients. Sixteen of these patients underwent an ALND. Five of this group had further nodal disease. Therefore, micrometastases and ITCs, detected only by IHC analysis of SNs, are associated with further positive nodes in the axilla in *up to 15%* of patients. However, more research is needed and IHC is not yet the standard procedure in most pathology departments.

### 3.3. Effect on Survival of Completion ALND

Completion ALND remains the gold standard for patients with a tumor-involved SN. ALND achieves regional control, but its effect on *survival* remains controversial. The *American College of Surgeons Oncology Group (*ACOSOG) Z0011 study randomized clinically node-negative patients who underwent SN biopsy and had 1 or 2 SN with metastases detected by H&E to ALND or no further axillary surgery [41]. Ineligibility criteria were SN metastasis detected by IHC, 3 or more SN positive, third field RT for nodal RT or accelerated partial breast irradiation (APBI). Both groups have tangent breast RT plus *systemic therapy* (which can be hormone or chemotherapy). The 446 patients with SN biopsy alone and 445 patients with SN biopsy plus ALND were similar in prognostic factors. Median followup is 6.2 years.  Table 4 summarizes the results.

So despite the widely held belief that ALND improves survival, no significant difference was recognized by this study of SN node-positive women. Although the study closed early because of low accrual/event rate, it is the largest phase III study of ALND for node-positive women, and it demonstrates no trend toward clinical benefit of ALND for patients with *limited* nodal disease *and given adjuvant systemic therapy*.

*Based on the above evidence, the authors of this paper believe that when the estimated risk of nonSN involvement is low enough, a completion ALND is not necessary. Even if there is further involved nonSN(s), they may be treated by systemic therapy or tangent field RT* which covers level I and some level II axillary nodes. The survival benefit from radiation is best explained by the prevention of an isolated loco-regional recurrence, which could serve as a source of fatal distant metastases and parallels the difference in the total incidence of distant metastases [42].

### 3.4. Important Aspects of a Standard Pathology Report

See Table 4.

## 4. Conclusion

We have summarized the studies and analyzed them as a whole to draw the following conclusions. Misleading studies due to small patient numbers and short followup have clouded the issue of poor outcome of pN1mi before. The authors of this paper felt that pN1mi patients have worse outcome than node-negative patients. Patients with micrometastases or ITC benefit from adjuvant systemic treatment as evident from de Boer et al. [21].

When the *estimated* risk of nonSN involvement is low enough, a completion ALND is not necessary. Even if there is further involved nonSN(s), they may be treated by systemic therapy or tangent field RT which covers level I and some level II axillary nodes. In the ACOSOG study, for clinically node-negative patients who underwent SN biopsy and had 1 or 2 SN with metastases detected by H&E, completion ALND would not affect local recurrence, OS or DFS [41]. Hence provided patients had *limited nodal disease and receive *adjuvant systemic treatment, completion ALND after SN biopsy is not warranted.

## Figures and Tables

**Table 1:** Definition of minimal pathologic lymph node involvement in American Joint Committee on Cancer (AJCC) Staging Manual, seventh edition (2010) [16, 17].

pN0(i−)	No regional lymph node metastases histologically, negative immunohistochemistry (IHC)
pN0(i+)	Malignant cells in regional lymph node(s) no greater than 0.2 mm (detected by H&E or IHC including isolated tumor cell clusters (ITC))
pN0(mol−)	No regional lymph node metastases histologically, negative molecular findings (RT-PCR, reverse transcriptase/polymerase chain reaction)
pN0(mol+)	Positive molecular findings (RT-PCR), but no regional lymph node metastases detected by histology or IHC
pN1	Micrometastases; or metastases in 1–3 axillary lymph nodes; and/or in internal mammary nodes with metastases detected by sentinel lymph node biopsy but not clinically detected
pN1mi	Micrometastases (greater than 0.2 mm and/or more than 200 cells, but none greater than 2.0 mm)

**Table 2:** Important studies on micrometastatic nodes in breast cancer.

Author	Study	Median FU	Patient number	Conclusion
Bulte et al. [18]	7 hospitals in Netherlands	3.8 years	503 pN0 38 pNmi	Local relapse—5(1.0%) versus 1(2.6%) Regional relapse—0% versus 0% Distant relapse—17(3.4%) versus 3(7.9%) Combined locoregional relapse—1(0.2%) versus 0% Combined regional and distant relapse—3(0.6%) versus 0% (n.s., see text for details)
Hainsworth et al. [19]	St Vincent's Hospital, Australia	6.6 years	31/343 occult node metastases found on IHC, plus 10 found on H&E	Among the 31 patients, presence of occult metastases in 2 or more nodes was associated with decreased DFS and OS (*P* < .05)
Truong et al. [20]	Surveillance Epidemiology and End Results database	7.3 years	62,551 pT1–2pN0-: 57,980 pN0, 1818 pNmi, 2753 pNmac >2 mm but <2 cm	10-year BCSS (82.3% versus 91.9%) and OS (68.1% versus 75.7%) in pNmi compared to pN0. (s.s.)
Colleoni et al. [29]	Italian medical oncology department	4.2 years	1959 pT1-3, pN0, pN1mi or pN0i+), or pN1a (single positive node) and M0	pN1mi/pN0i+ versus pN0 disease: HR = 1.58; 95% CI, 1.01 to 2.47; *P* = .047 for DFS; HR = 1.94; 95% CI, 1.04 to 3.64; *P* = .037 for distant metastases.
de Boer et al. [21]	Dutch cohort study of all centers in Netherlands (MIRROR study)	5.1 years	(a) 856 Nmi/ITC without adjuvant therapy, (b) 995 Nmi/ITC with adjuvant therapy, (c) 856 node-negative	Disease events: (a) for Nmi: HR 1.56 (95% CI:1.15-2.13; for ITC:HR 1.50 (95% CI:1.15-1.94) (b) HR 0.57 (95% CI:0.45-0.73)
Houvenaeghel et al. [33]	A French center	—	SN involvement in 388 times (55.4%) by H&E, 312 times by IHC	May omit additional ALND for pT1a and pT1b tumors, and pT1a-c tumors corresponding to tubular, colloidal or medullary cancers

ALND: axilllary lymph node dissection; DFS: disease-free survival; FU: followup; n.s.: statistically nonsignificant; OS: overall survival; SNB: sentinel node biopsy; s.s.: statistically nonsignificant.

**Table 3:** Summary of results of American College of Surgeons Oncology Group (ACOSOG) Z0011 study.

	ALND	SN biopsy	*P* values
5-year in-breast recurrence	3.7%	2.1%	.16
5-year nodal recurrence	0.6%	13%	.44
5-year overall survival	91.9%	92.5%	.24
5-year disease-free survival	82.2%	83.8%	.13

**Table 4:** Summary of important aspects of a standard pathology report—this would enable oncologists to make individual decision on management, including completion ALND and adjuvant systemic treatment.

Primary tumor	
Multifocal or multicentric	
In-situ component	
Grade	
Necrosis	
Lymphovascular invasion	
Margin	
Histologic subtype	
Immunohistochemical HER2, ER, PR	
Involvement of nipple or skeletal muscle	
Abnormalities of surrounding breast tissue	
NODE	
Volume or size of nodal metastasis	
Location within the node	
Extranodal extension	
Number of involved sentinel node	
Number of involved nonsentinel node	
Total number of sentinel node	
Total number of nodes on axillary dissection, if performed	
